# Differential physiological responses and transcriptome co-expression networks of salt-tolerant and salt-sensitive foxtail millet (*Setaria italica* (L.)) under salt stress

**DOI:** 10.3389/fpls.2026.1772695

**Published:** 2026-02-16

**Authors:** Min Liu, Zhi-Wei Wang, Song Hou, Ru-Mei Tian, Kun Xie, Jing Bai, Yun-Zhe Cong, Yongyi Yang, Wei Liu

**Affiliations:** 1 National Saline-Alkali Tolerant Crop Germplasm Resources Nursery (Dongying), Shandong Crop Germplasm Resources Bank, Institute of Crop Germplasm Resources, Shandong Academy of Agricultural Sciences, Jinan, China; 2 Agricultural College, Henan University of Science and Technology, Luoyang, China

**Keywords:** co-expression network, foxtail millet, salt-sensitive, salt-tolerant, *Setaria italica*, WGCNA

## Abstract

**Introduction:**

Salt stress severely limits crop productivity by disrupting ion homeostasis and cellular metabolism. Foxtail millet (*Setaria italica* (L.)), a stress-resilient cereal, exhibits marked natural variation in salt tolerance, yet the regulatory mechanisms underlying this divergence remain unclear.

**Methods:**

Here, we integrated physiological assessments, time-course transcriptome profiling, and weighted gene co-expression network analysis (WGCNA) to dissect salt stress responses in a salt-tolerant accession (SDT80) and a salt-sensitive accession (SDS81). Key indicators of ion balance and oxidative damage were measured, and co-expression modules and hub genes associated with salt tolerance were identified.

**Results:**

Under salt stress, SDT80 maintained lower Na^+ ^ accumulation, a more stable Na^+ ^/K^+ ^ ratio, and reduced membrane lipid peroxidation compared with SDS81. Transcriptomic analyses revealed dynamic and genotype-dependent expression patterns: SDT80 preferentially activated abiotic stress-related pathways, whereas SDS81 showed enrichment in processes linked to photosynthetic inhibition and cellular injury. WGCNA identified 23 co-expression modules, among which two key modules were strongly correlated with treatment duration, ion contents, and oxidative stress indices. Hub-gene analysis suggested that one module functions as a regulatory core integrating transcriptional control, calcium signaling, and metabolic adjustment, while the other is mainly involved in detoxification, energy metabolism, and cell wall remodeling.

**Discussion:**

Collectively, our integrative network analyses indicate that salt tolerance in foxtail millet arises from coordinated regulatory networks coupling ion homeostasis, stress signaling, and metabolic reprogramming rather than single-gene effects, providing candidate targets for improving salt tolerance in millet and other crops.

## 1 Introduction

Salt stress is one of the most pervasive abiotic stresses limiting crop productivity worldwide (Majeed and Muhammad, 2019). More than 800 million hectares of land are affected by salinization, and the affected area continues to expand due to irrigation mismanagement, climate change, and soil degradation (Shahid et al., 2018; Singh, 2022). Excessive salt imposes both ionic and osmotic challenges on plants, leading to growth inhibition, metabolic dysfunction, membrane injury, and ultimately severe yield penalties (Ma et al., 2025). The complexity of salt stress responses arises from the interplay among osmotic stress, ionic imbalance, oxidative damage, and hormonal signaling (Munns and Tester, 2008; Xiao and Zhou, 2022). Thus, elucidating the molecular mechanisms underlying plant salt tolerance is essential for improving crop resilience and securing global food production.

A hallmark physiological response to salt stress is the rapid accumulation of reactive oxygen species (ROS), which leads to membrane lipid peroxidation and cellular damage (Peláez-Vico et al., 2024). Malondialdehyde (MDA), a major product of lipid peroxidation, is widely used as a sensitive indicator for assessing oxidative injury under salt stress conditions (Gill and Tuteja, 2010). The degree of MDA accumulation often correlates with the plant’s salt sensitivity, making it an important physiological trait for evaluating tolerance mechanisms. Concurrently, the maintenance of ionic homeostasis—particularly a low cytosolic Na^+^/K^+^ ratio—is a well-established determinant of salt tolerance. Key ion transport systems, including the SOS pathway components (SOS1–SOS3) (Ali et al., 2023), HKT transporters (Ali et al., 2021), NHX antiporters (Abdelrady et al., 2025), and various K^+^ channels and transporters, function synergistically to exclude Na^+^ from the cytosol, sequester Na^+^ into vacuoles, and maintain K^+^ uptake under stress (Munns and Tester, 2008; Shabala and Cuin, 2008; Ma et al., 2025). Despite advances in understanding these pathways, how these processes are dynamically orchestrated across different time scales, and how they differ between tolerant and sensitive genotypes, remain incompletely understood.

Foxtail millet (*Setaria italica* (L.)) is an emerging model C4 crop with exceptional drought and stress tolerance, a small genome, abundant genetic resources, and a short life cycle (Rathinapriya et al., 2020). Its natural variation in abiotic stress responses makes it a valuable system for dissecting salt tolerance mechanisms. Although several studies have reported salt-responsive genes or pathways in foxtail millet, most existing work focuses on single time points or individual genes (Chu et al., 2025; Zhang et al., 2025a). A comprehensive understanding of genotype-specific and time-dependent transcriptional reprogramming in response to salt stress is still lacking. Particularly, it remains poorly understood how foxtail millet coordinates oxidative stress mitigation and ionic homeostasis across different stages of salt adaptation, and which gene networks underpin the contrasting responses of tolerant versus sensitive genotypes.

Time-series transcriptome analysis is critical for dissecting the temporal dynamics of gene expression during plant stress responses (Wang et al., 2025c). Compared with static single-time-point analyses, high-resolution time-series profiling enables comprehensive capture of early signaling events, subsequent transcriptional reprogramming, and late-stage adaptive responses (Zhao et al., 2023; Kuang et al., 2025). When integrated with weighted gene co-expression network analysis (WGCNA), it becomes possible to identify modules of co-expressed genes that are tightly associated with key physiological traits, such as MDA, Na^+^/K^+^ ratio, or ion transport activity. WGCNA further enables prioritization of hub genes that may serve as central regulators in stress-responsive pathways (Langfelder and Horvath, 2008; Wang et al., 2025a). This systems-level view provides deeper biological insights than traditional DEG-based analyses and has proven powerful in unraveling complex traits including salt tolerance in multiple crops (Wang et al., 2025b).

To address the current knowledge gaps, we conducted a comprehensive time-series RNA-seq analysis (0 h, 3 h, 6 h, 12 h, 24 h, 48 h, 72 h) in a salt-tolerant and a salt-sensitive foxtail millet accessions under NaCl stress. By integrating transcriptome dynamics with WGCNA and trait association analyses, we constructed co-expression networks related to ion transport processes and membrane damage (MDA). This approach enabled us to (i) characterize temporal differences in transcriptional responses between contrasting genotypes, (ii) identify key gene modules associated with oxidative injury and ionic homeostasis, and (iii) pinpoint candidate hub genes potentially governing foxtail millet salt tolerance. Our results provide a comprehensive regulatory landscape of salt stress adaptation in foxtail millet and offer valuable genetic resources for improving salt tolerance in cereal crops.

## 2 Materials and methods

### 2.1 Plant materials, growth conditions and treatments

Two foxtail millet landraces with contrasting salt tolerance were used in this study. The salt-tolerant accession, designated SDT80 (Shandong Tolerant 80), and the salt-sensitive accession, SDS81 (Shandong Sensitive 81), were both collected from local traditional accessions cultivated in Shandong Province, China. Uniformly sized and vigorously germinated seeds were selected and sown in 96-well hydroponic trays, with two seeds per well. The growth medium consisted of half-strength Murashige and Skoog (1/2 MS) nutrient solution. Plants were grown in a controlled growth chamber under the following conditions: temperature at 26°C, photoperiod of 16 hours light/8 hours dark, and relative humidity of 70%. After 15 days of cultivation, salt stress treatments were applied using 150 mM NaCl. At 0 h, 3 h, 6 h, 12 h, 24 h, 48 h, and 72 h after salt treatment, shoot tissues were harvested for physiological assays and RNA-seq analysis. Each treatment was performed with three biological replicates.

### 2.2 Measurement of physiological parameters

Measuring the Na^+^ and K^+^ content in plants were estimated by following the method of Jezek et al (Jezek et al., 2014) with little modification. A 0.2 g sample was placed in a digestion vessel, and 10 mL of concentrated nitric acid was added. The vessel was loosely capped and allowed to stand at room temperature for 1 hour. After securely capping the vessel, the sample was digested using a microwave digestion system. Upon cooling, the vessel was carefully opened to release the gases, and the inner cap was rinsed with deionized water. The solution was subjected to 5 minutes of ultrasonic degassing, diluted to a final volume of 20 mL with deionized water, and thoroughly mixed. Blank controls were processed concurrently. The cation concentrations of Na^+^ and K^+^ were determined using an inductively coupled plasma mass spectrometer (ICP-MS, Agilent 7800). Standard curves were prepared using element standards sourced from the National Standard Material Center. Each batch included both reagent and experimental blanks. Finally, the ion content in the sample was calculated based on the results.

MDA contents represent the lipid peroxidation which was measured by following Senthilkumar et al. procedure (Senthilkumar et al., 2021) with slight modifications. The sample was ground into a powder using liquid nitrogen, and approximately 0.1 g of tissue was ground in liquid nitrogen and homogenized in 0.5 mL of phosphate-buffered saline (PBS) on ice. The homogenate was centrifuged at 8000 × g for 10 min at 4°C, and the supernatant was collected and kept on ice. For the assay, 60 μL of the supernatant was mixed with 180 μL of 0.5% thiobarbituric acid (TBA) solution in a 1.5 mL microcentrifuge tube. The mixture was incubated at 95°C for 30 min in sealed tubes, then rapidly cooled on ice. After centrifugation at 10,000 × g for 10 min at 25°C, 200 μL of the clear supernatant was transferred to a 96-well microplate. Absorbance of the supernatant was measured at 532 nm and 600 nm, and the MDA concentration was calculated from the absorbance difference (A_532_ – A_600_) using an extinction coefficient of 1.56 × 10^5^ L·mol^-1^·cm^-1^.

### 2.3 RNA extraction, library preparation, and sequencing

Total RNA was extracted using the FreeZol Reagent kit (R711, Vazyme, China), and RNA purity and integrity were confirmed via NanoDrop 2000 spectrophotometry (Thermo Scientific, USA) and agarose gel electrophoresis. Polyadenylated mRNA was isolated with Oligo (dT) magnetic beads, fragmented, and reverse transcribed to synthesize double-stranded cDNA. The cDNA was end-repaired, A-tailed, and ligated with MGI-specific adapters, followed by PCR amplification and purification. Final libraries were circularized to generate single-stranded circular DNA (ssCir DNA) for DNA nanoball (DNB) formation. Libraries were sequenced on the DNBSEQ platform (MGI Tech, China) following the manufacturer’s protocols.

### 2.4 Gene expression quantification and differential expression analysis

Raw sequencing reads were processed with Fastp (Chen, 2023) to trim adapters, filter low-quality reads, and remove sequences containing more than 5% ambiguous nucleotides. Clean reads were aligned to the foxtail millet reference genome (Yugu1-T2T) (He et al., 2024) using HISAT2 (Zhang et al., 2021). Gene expression levels were quantified and normalized as transcripts per million (TPM), and genes with TPM < 1 across all samples were excluded to reduce background noise. Differentially expressed genes (DEGs) were identified using DESeq2 (Love et al., 2014) with size factor normalization and a parametric fit model. Genes were considered significantly differentially expressed if they met the criteria of an adjusted P-value < 0.05 (Benjamini–Hochberg correction) and an absolute |log_2_FoldChange| ≥ 1. Functional annotation was performed using eggNOG-mapper v2 (Cantalapiedra et al., 2021). based on the eukaryotic database with default parameters.

### 2.5 GO and KEGG enrichment analysis

Enrichment analysis of Gene Ontology (GO) terms and Kyoto Encyclopedia of Genes and Genomes (KEGG) pathways was conducted using the clusterProfiler package (Xu et al., 2024), using all expressed genes (TPM ≥ 1) as the background. Only terms with adjusted P-values < 0.05 were considered significantly enriched. Data visualization was performed using the ggplot2 package (Villanueva and Chen, 2019).

### 2.6 WGCNA and functional annotation

WGCNA was conducted using RNA-seq expression data from foxtail millet shoot tissues subjected to NaCl treatment at multiple time points across two accessions (Wang et al., 2025a). To construct a robust co-expression network, we first retained genes with TPM > 1 in at least one sample. Differential expression was then evaluated using DESeq2 based on the corresponding count data (adjusted P-value < 0.05 and |log2FoldChange| ≥ 1). DEGs identified from all relevant comparisons were merged (union) to generate the final DEG set, and the TPM expression matrix of this gene set across all samples was used as the input for subsequent WGCNA network construction and module detection. Next, the soft-thresholding power was determined using the pickSoftThreshold function in the WGCNA R package (Langfelder and Horvath, 2008) to approximate a scale-free topology, and a power of 11 was selected. Using this power, Pearson correlations were soft-thresholded to construct the adjacency matrix, and the topological overlap matrix (TOM) was then calculated to quantify network connectivity. Hierarchical clustering of TOM-based dissimilarity was performed to identify gene co-expression modules, with a minimum module size of 30 and deepSplit set to 2. Modules with highly correlated eigengenes (correlation > 0.75) were merged using a mergeCutHeight of 0.25. Finally, module eigengenes were computed and correlated with salt-stress treatments and associated physiological traits to identify modules significantly linked to salt-stress responses. Hub genes within key modules were identified based on intramodular connectivity and visualized using Cytoscape software (Shannon et al., 2003). Conserved protein domains of hub genes were verified using Batch CD-Search (Marchler-Bauer et al., 2017).

### 2.7 Cross-network module correspondence and conservation analysis

To investigate the conservation and rewiring of gene co-expression networks between the two foxtail millet cultivars SDT80 and SDS81, WGCNA was independently conducted on the SDT80 dataset, the SDS81 dataset, and the combined dataset (SDT80&SDS81, total). Module assignment tables were generated for each analysis, and only genes shared by all three datasets were retained for cross-network comparison.

Module correspondence among the three networks was examined at the gene level by integrating the three module assignment tables according to gene IDs. To visualize large-scale module splitting, merging, and rewiring events, Sankey diagrams were constructed using the networkD3 R package^1^, in which module memberships were directionally connected from SDT80 to total and subsequently to SDS81. The width of each flow represents the number of genes shared between corresponding module pairs, thereby illustrating module conservation and network remodeling across cultivars.

To quantitatively assess the significance of module conservation between accession-specific networks, pairwise module overlap analysis was further performed between SDT80 and SDS81 using the GeneOverlap R package^2^. For each module pair, Fisher’s exact tests (Mehta and Patel, 1983) based on the hypergeometric distribution were applied to determine whether the observed gene overlap was significantly greater than expected by chance. Module pairs with P < 0.001 were considered significantly conserved and were used to identify core conserved modules and accession-specific rewiring patterns.

## 3 Results

### 3.1 Physiological responses of foxtail millet under salt stress

**Figure 1:**
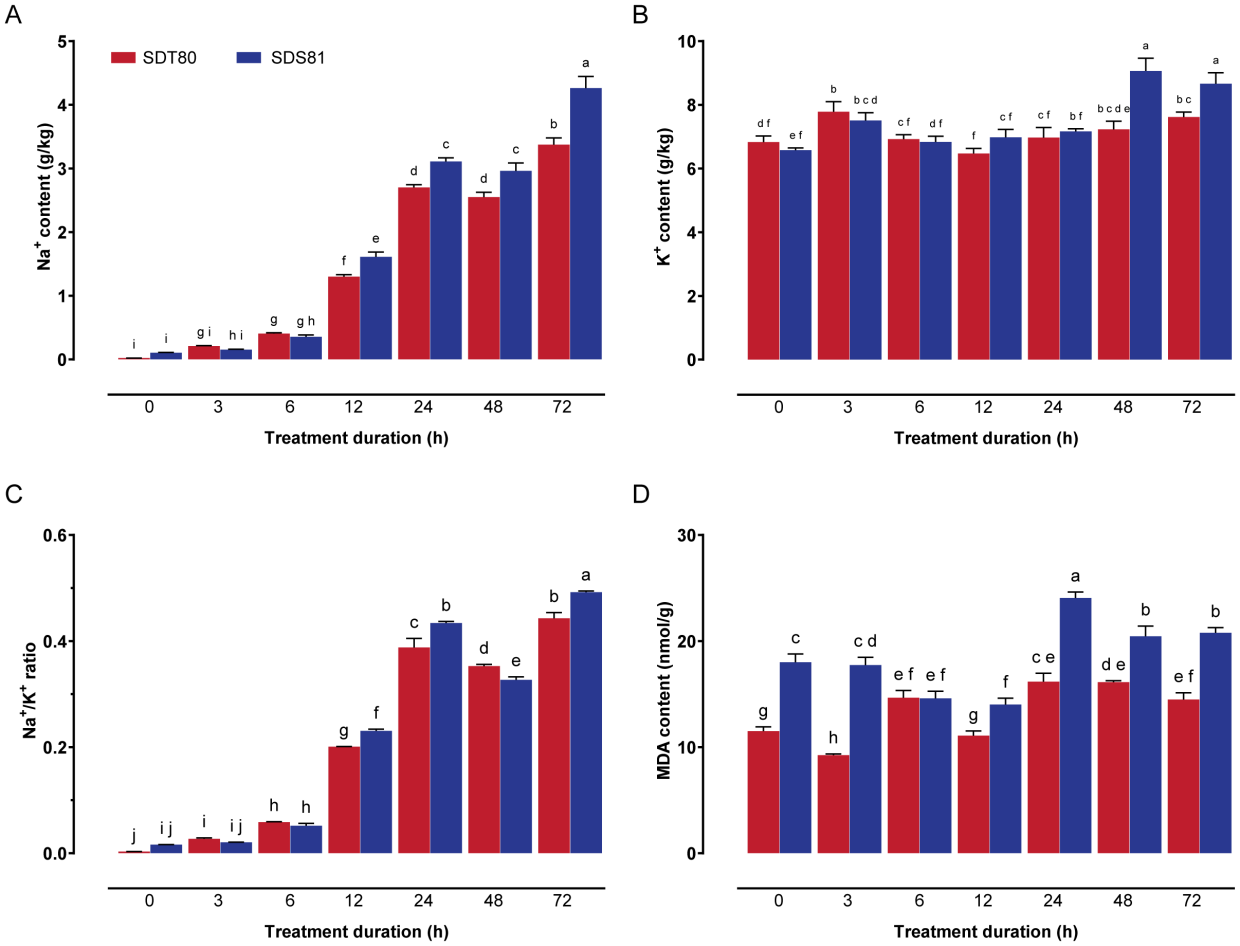
Physiological responses of foxtail millet under salt stress. **(A)** Na^+^ content in the shoots of SDT80 and SDS81 at different salt treatment durations, **(B)** K^+^ content in the shoots of SDT80 and SDS81 at different salt treatment durations, **(C)** Na^+^/K^+^ ratio in the shoots of SDT80 and SDS81 at different salt treatment durations, **(D)** MDA content in the shoots of SDT80 and SDS81 at different salt treatment durations. Different letters indicate significant differences between groups (*P* < 0.05).

To evaluate ion toxicity in plants, 15-day-old foxtail millet (SDT80 and SDS81) seedlings were treated with 150 mM NaCl for 0 h, 3 h, 6 h, 12 h, 24 h, 48 h, and 72 h. The results indicated a significant increase in Na^+^ content in both accessions as the salt treatment duration increased. Notably, from 12 h onward, the Na^+^ content in the salt-sensitive accessions SDS81 was significantly higher than that in the salt-tolerant accession SDT80, indicating defective regulation of Na^+^ homeostasis in SDS81 and consequently more severe ionic toxicity (**Figure 1A**). Regarding K^+^ content, both accessions exhibited a significant increase at 3 h, with SDS81 showing a marked elevation in K^+^ content at 48 h. In contrast, SDT80 did not exhibit significant changes in K^+^ content at other time points. Comparison of K^+^ content between SDT80 and SDS81 revealed no significant differences during the early stages of salt treatment. However, at 48 h and 72 h, SDS81 exhibited significantly higher K^+^ content compared to SDT80 (**Figure 1B**). The Na^+^/K^+^ ratio showed an overall increasing trend and exhibited a decrease at 48 h, which was primarily attributable to a transient increase in K^+^ content in SDS81 rather than reduced Na^+^ accumulation. At this point, SDT80 had a significantly higher Na^+^/K^+^ ratio than SDS81. At 12 h, 24 h, and 72 h, SDT80 exhibited significantly lower Na^+^/K^+^ ratios compared to SDS81, indicating that SDT80 is more effective in regulating Na^+^ and K^+^ accumulation, thereby reducing ion toxicity (**Figure 1C**).

To assess membrane lipid peroxidation under salt stress, we measured MDA content of the shoot of foxtail millet. The results showed that MDA content in SDT80 initially decreased, followed by an increase, then a subsequent decrease and stabilization. In contrast, SDS81 displayed a decrease followed by an increase and then a decrease. Except at the 6-hour time point, MDA content in SDT80 was significantly lower than in SDS81 at all other time points (**Figure 1D**), suggesting that the salt-sensitive accession SDS81 is more susceptible to membrane damage under salt stress, which may contribute to reduced salt tolerance.

### 3.2 Differential gene expression and enrichment analysis of foxtail millet under salt stress

To investigate the molecular response of foxtail millet to salt stress at different treatment time points, total RNA was extracted from shoot tissues collected at seven time points (0–72 h) from both accessions, with three biological replicates per time point. and subjected to high-throughput transcriptome sequencing. A total of 1,958,113,370 raw reads were generated across all samples. After stringent quality control—including removal of adapter sequences, poly-A/T tails, and low-quality reads—a total of 1,957,549,616 clean reads were retained. Over 39 million high-quality reads were generated per sample, with Q20 and Q30 scores exceeding 98% and 94%, respectively. The clean reads were mapped to the foxtail millet reference genome (He et al., 2024) with mapping ratios greater than 97.64%, demonstrating the high fidelity of sequencing (**Supplementary Table 1**).

**Figure 2:**
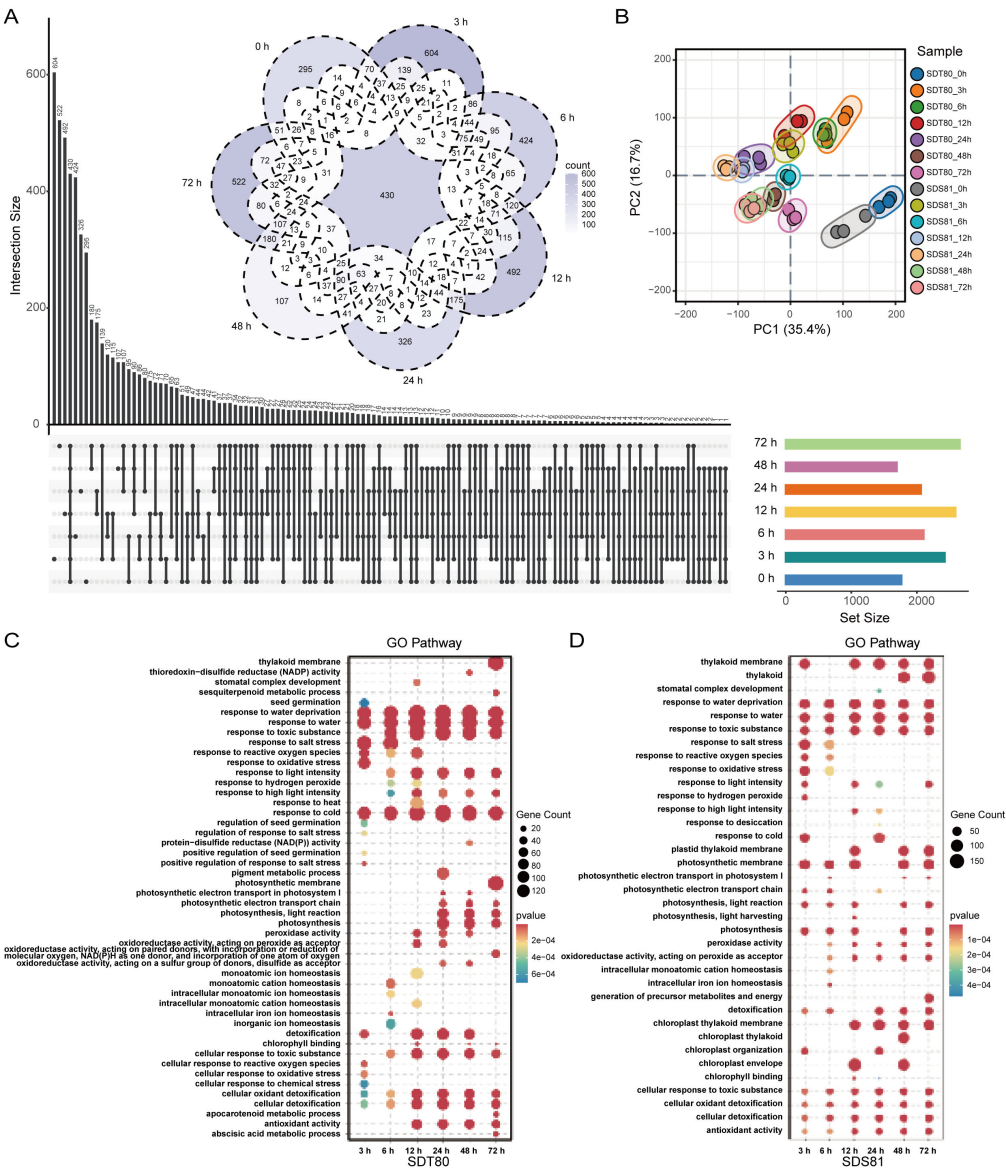
Comparative analysis and functional enrichment of differentially expressed genes under salt stress. **(A)** Intersection and specificity analysis of gene sets, showing the distribution of DEGs across treatments. **(B)** PCA analysis of different treatment samples. **(C)** GO enrichment analysis of DEGs under different salt treatment durations of SDT80. **(D)** GO enrichment analysis of DEGs under different salt treatment durations of SDS81.

A total of 15,373 differentially expressed genes were identified from pairwise comparisons among the samples. In accession SDT80, the number of DEGs initially increased and then decreased with the extension of treatment duration, reaching a maximum at 24 h. In contrast, accession SDS81 exhibited a fluctuating pattern in DEG numbers over time yet also showed the highest number at 24 h. Comparison between the two accessions revealed no clear temporal trend in DEG numbers; nevertheless, the largest difference was observed at 72 h (**Supplementary Table 2**). Compared with SDT80, SDS81 displayed distinct transcriptomic responses to salt stress across all examined time points (0 h, 3 h, 6 h, 12 h, 24 h, 48 h, and 72 h). Specifically, 295, 604, 424, 492, 326, 107, and 522 DEGs were uniquely identified at these respective time points. In total, 430 DEGs were commonly detected across all seven time points. Notably, the number of specific DEGs peaked at 3 h, followed by 72 h, whereas the lowest number was observed at 48 h, indicating dynamic temporal changes in gene expression during the salt stress response of SDS81 (**Figure 2A**). Principal component analysis (PCA) revealed clear separation between SDT80 and SDS81 across all time points under salt stress, with good clustering among the three biological replicates. The first two principal components (PC1 and PC2) explained 35.4% and 16.7% of the total variance, respectively. The clustering differences among genotypes and time points further reflected their divergent transcriptional responses to salt stress (**Figure 2B**).

GO and KEGG enrichment analyses were performed to explore the biological functions of the DEGs between SDT80 and SDS81 under salt stress. GO enrichment analysis revealed that the DEGs in SDT80 were predominantly enriched in biological processes related to plant hormone signaling regulation and ion homeostasis (**Supplementary Figure 1**), KEGG pathway analysis further showed that these DEGs were mainly involved in plant hormone signal transduction, photosynthesis, and carbon metabolism pathways (**Supplementary Figure 2**). In contrast, DEGs in SDS81 were primarily enriched in photosynthesis-related processes as well as in the maintenance of chloroplast and thylakoid structure and function according to GO analysis (**Supplementary Figure 3**), while KEGG analysis indicated that these DEGs were mainly associated with plant hormone signal transduction, reactive oxygen species metabolism, and detoxification-related pathways (**Supplementary Figure 4**). Further enrichment analysis of salt stress–related GO terms showed that the differentially expressed genes in the salt-tolerant accession SDT80 were predominantly enriched in abiotic stress response processes, such as response to salt stress, response to water, and response to cold (**Figure 2C**). This suggests that SDT80 can rapidly activate signaling pathways and antioxidant defenses at the early stages of salt stress, thereby maintaining cellular homeostasis and enhancing salt tolerance. In contrast, the enriched terms in the salt-sensitive accession SDS81 were more focused on processes related to cell death and protein degradation. Notably, in the later stages of stress, terms such as thylakoid and chloroplast thylakoid membrane were significantly enriched, indicating that SDS81 experiences severe cellular damage under prolonged salt stress and requires enhanced membrane system repair to cope with stress-induced damage (**Figure 2D**).

### 3.3 WGCNA of foxtail millet under salt stress

**Figure 3:**
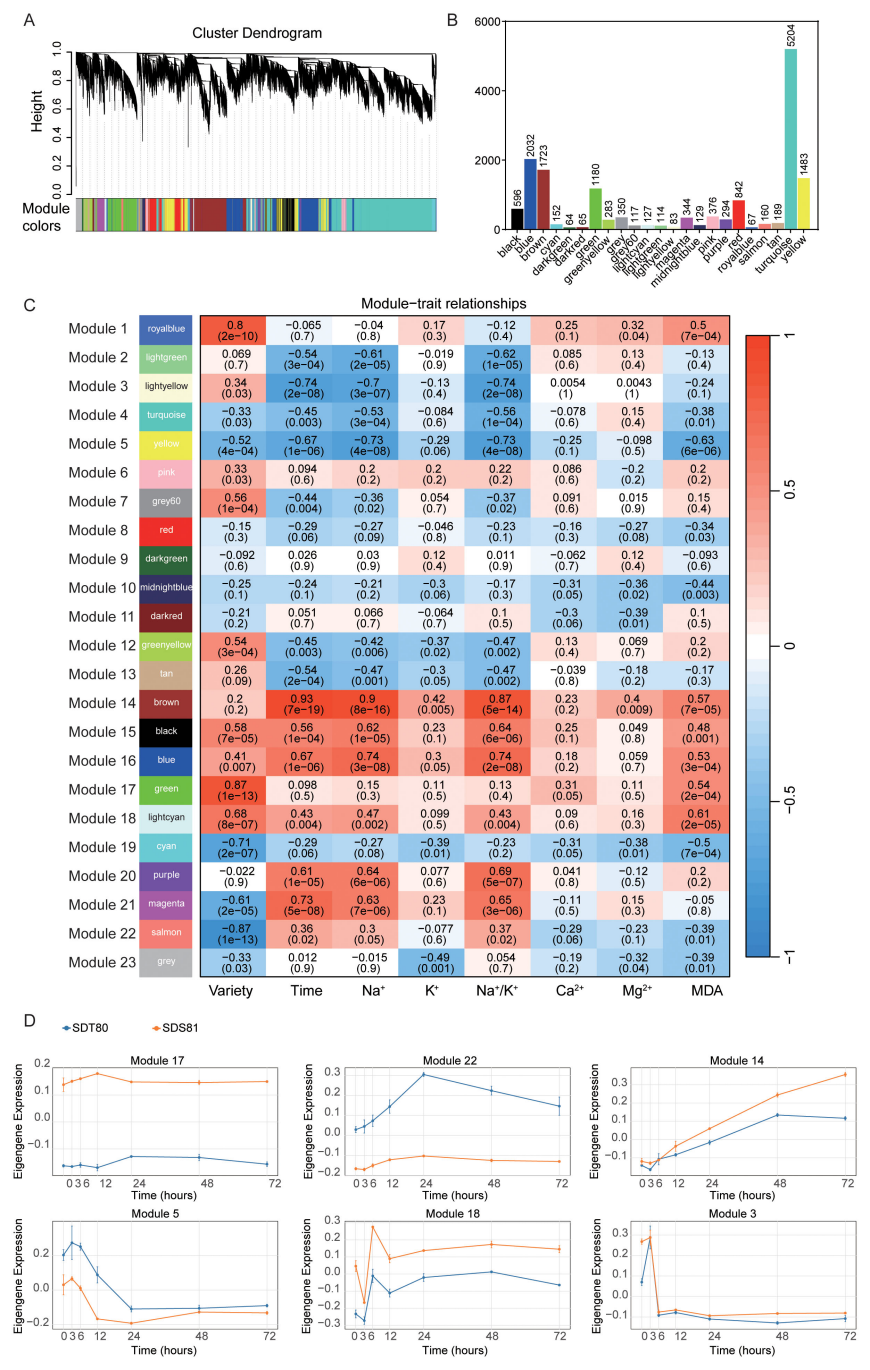
WGCNA of DEGs under salt stress. **(A)** Gene clustering and module recognition in foxtail millet. Different modules are marked with different colors. **(B)** Number of genes in each module. **(C)** Correlation analysis between modules and variety, treatment time, and physiological indicators. The numbers within the modules represent correlation coefficients, and the values in parentheses indicate *p*-values. **(D)** Temporal expression patterns of module eigengenes in response to salt stress. Line plots show the time-course changes of eigengene expression for six representative co-expression modules under salt treatment in SDT80 (blue) and SDS81 (orange).

To further investigate differences in salt stress regulatory mechanisms between the two accessions, WGCNA was conducted using differentially expressed genes, resulting in the identification of 23 co-expression modules (**Figure 3A**). The module numbers are used solely for identification and do not indicate priority. Module 4 contained the largest number of genes (5,204), whereas Module 9 contained the fewest (64) (**Figure 3B**).

Module–trait relationship analysis showed that Module 17 was most positively correlated with accession differences, while Module 22 exhibited the strongest negative correlation (**Figure 3C**). Consistently, genes in Module 17 were expressed at higher levels in SDS81 than in SDT80, whereas the opposite trend was observed for Module 22 (**Figure 3D**). Module 14 showed the strongest positive correlation with treatment duration, whereas Module 3 showed the strongest negative correlation (**Figure 3C**), accordingly, Module 14 expression increased over time in both accessions, while Module 3 expression declined (**Figure 3D**).

Regarding ion contents and physiological traits, Module 14 was strongly positively correlated with Na^+^ content, K^+^ content, Na^+^/K^+^ ratio, and Mg^2+^ content, whereas Module 5 showed strong negative correlations with Na^+^ content and the Na^+^/K^+^ ratio. Module expression dynamics were consistent with ion content trends (**Figure 1**). Module 17 showed the strongest positive correlation with Ca^2+^ content, while Module 10 and Module 19 were negatively correlated. For oxidative stress indices, Module 18 was most positively correlated with MDA levels, whereas Module 5 showed a strong negative correlation (**Figure 3C**).

GO (**Supplementary Table 3**) and KEGG (**Supplementary Table 4**) enrichment analyses of five key modules revealed distinct functional profiles. Module 14 was enriched in stress and immune responses, secondary metabolism, redox activity, and transmembrane transport, with KEGG pathways including plant–pathogen interaction, MAPK signaling, brassinosteroid biosynthesis, and terpenoid metabolism. Module 17 was enriched in RNA polymerase II–related transcriptional regulation and metabolic pathways such as fatty acid, β-alanine, and branched-chain amino acid metabolism. Module 3 was enriched in metal ion homeostasis and iron response, consistent with KEGG enrichment in mineral absorption and ferroptosis. Module 18 was associated with stress and bacterial response, while Module 5 was enriched in secondary cell wall biogenesis, phenylpropanoid and flavonoid biosynthesis, and lipid metabolism.

### 3.4 Comparative analysis of co-expression network divergence in ion transport–related modules between two accessions under salt stress

**Figure 4:**
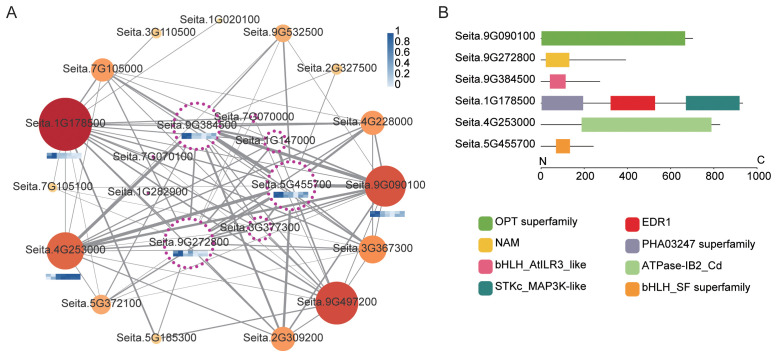
Analysis of key genes in Module 3. **(A)** Integrated co-expression network and expression heatmap of ion transport–related genes. Solid circles represent functional genes, and hollow dashed circles denote transcription factors. Node size and color reflect gene importance within the network. Edge thickness indicates the strength of co-expression. Heatmaps show min–max normalized and log-transformed expression levels, with the upper and lower rows representing SDT80 and SDS81, respectively, across NaCl treatment at 0 h, 3 h, 6 h, 12 h, 24 h, 48 h, and 72 h, darker blue indicates higher expression. **(B)** Conserved domain analysis of key genes. Lines represent protein sequences, and colored boxes indicate different conserved domains.

To elucidate the functional roles of the core modules identified by WGCNA in foxtail millet responses to salt stress, conserved domain analysis was performed for the hub genes in each module, and their expression patterns were compared between the salt-tolerant accession SDT80 and the salt-sensitive accession SDS81 at different time points. Module 3 was annotated as an early-response module associated with signal perception and ion homeostasis regulation, and was enriched in several transcription factors (**Figure 4A**), including NAM/NAC (Seita.9G272800) and bHLH family members (Seita.9G384500 and Seita.5G455700) (**Figure 4B**; **Supplementary Table 5**). Under control conditions, these genes exhibited lower expression levels in SDT80 than in SDS81, however, following salt treatment, they were rapidly upregulated in SDT80 and showed significantly higher expression than in SDS81. Subsequently, their expression levels declined in both accessions, accompanied by slight fluctuations (**Figure 4A**; **Supplementary Figure 5**). The gene ID Seita.1G178500, which contains an STKc_MAP3K-like/EDR1 domain, was markedly upregulated in SDT80 at the early stage of salt treatment and then maintained relatively stable expression, whereas only minor expression changes were observed in SDS81. In addition, two genes putatively involved in transmembrane ion transport were identified, namely Seita.4G253000, harboring an ATPase-IB2_Cd domain, and Seita.9G090100, containing an OPT superfamily domain (**Figure 4B**; **Supplementary Table 5**). Seita.4G253000 maintained relatively high expression levels at the late stage of salt treatment in both accessions. In contrast, Seita.9G090100 displayed a transient induction followed by repression in SDT80, whereas its expression in SDS81 showed an overall continuous decline, with a slight recovery at the final stage of treatment (**Figure 4A**; **Supplementary Figure 5**).

### 3.5 Comparative analysis of co-expression network divergence in Module 14 between two accessions under salt stress

**Figure 5:**
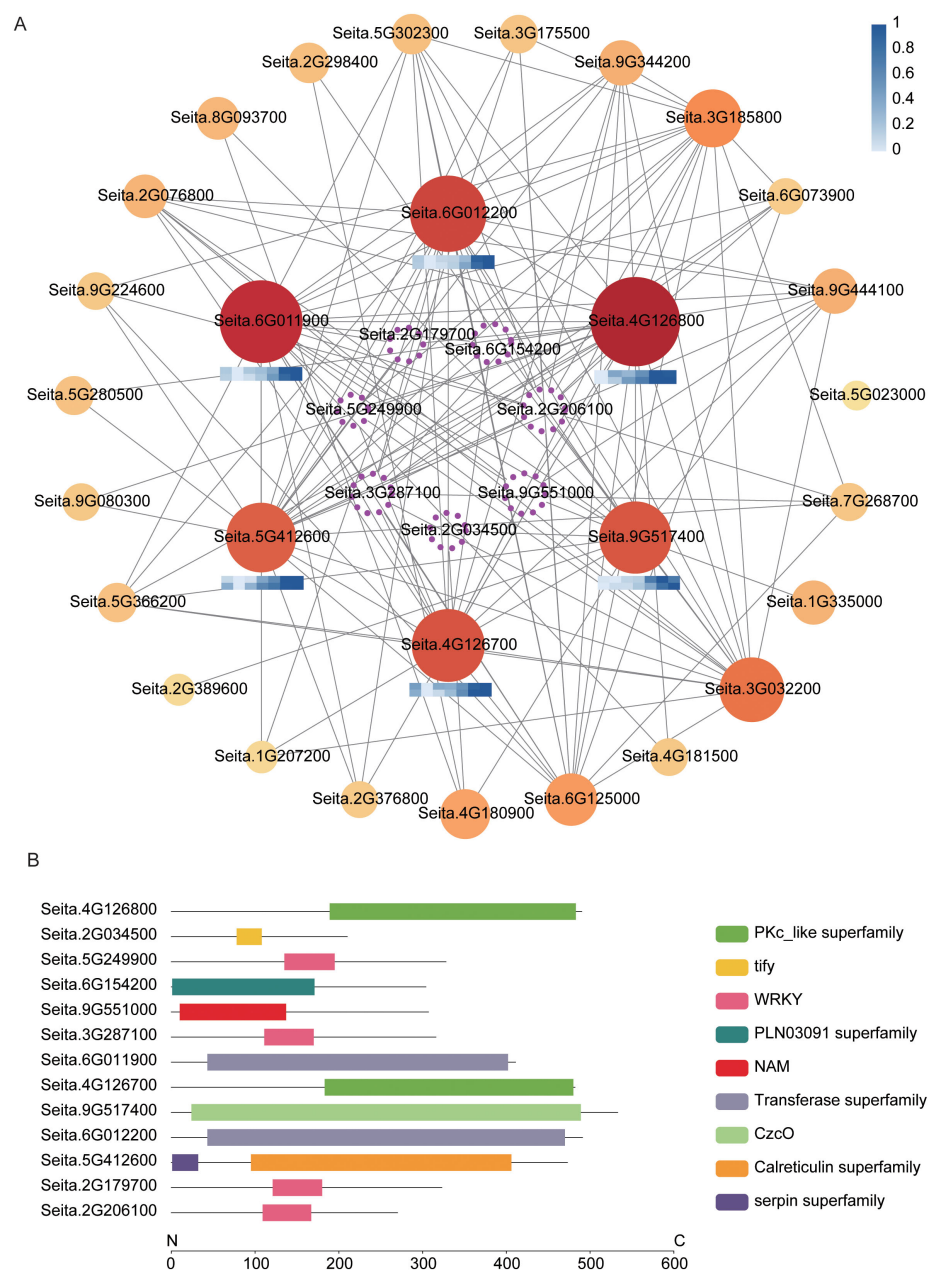
Analysis of key genes enriched in GO:0080134 within Module 14. **(A)** Integrated co-expression network and expression heatmap of key genes. Solid circles represent functional genes, and hollow dashed circles denote transcription factors. Node size and color reflect gene importance within the network. Edge thickness indicates the strength of co-expression. Heatmaps show min–max normalized and log-transformed expression levels, with the upper and lower rows representing SDT80 and SDS81, respectively, across NaCl treatment at 0 h, 3 h, 6 h, 12 h, 24 h, 48 h, and 72 h, darker blue indicates higher expression. **(B)** Conserved domain analysis of key genes. Lines represent protein sequences, and colored boxes indicate different conserved domains.

Module 14 represents a central regulatory hub for salt tolerance in this study and is characterized by a high enrichment of transcriptional regulators and signal transduction–related kinases. Analysis of the significantly enriched GO term GO:0080134 (regulation of response to stress) revealed that this module comprises seven key transcription factors and six functional genes, highlighting its pivotal role in coordinating salt stress responses (**Figure 5**).

Among the transcription factors, Seita.2G034500 belongs to the tify family and is primarily involved in hormone signal transduction, particularly in the jasmonic acid signaling pathway and stress responses (**Figure 5B**; **Supplementary Table 6**). Seita.6G154200 is a member of the MYB family, which plays important roles in the regulation of secondary metabolism, growth and development, and environmental stress responses. Seita.9G551000, belonging to the NAC family, is widely implicated in organ development, senescence, and responses to diverse biotic and abiotic stresses, and shows co-expression with WRKY genes. In addition, four transcription factors containing WRKY domains (Seita.2G179700, Seita.2G206100, Seita.3G287100, and Seita.5G249900) were identified in this module. The WRKY family is recognized as a core driver of plant stress responses, integrating both ABA-dependent and ABA-independent signaling pathways (**Figure 5B**; **Supplementary Table 6**).

In terms of signaling and functional genes, the presence of PKc_like domain–containing protein kinases (Seita.4G126700 and Seita.4G126800), together with the calreticulin domain–containing gene Seita.5G412600, further supports a central role of the Module 14 in calcium signaling cascades and downstream phosphorylation events. Specifically, Seita.4G126700 exhibited an expression pattern characterized by an initial decrease followed by an increase during salt treatment, with higher expression levels in SDT80 than in SDS81 at most time points. Seita.4G126800 showed higher expression in SDT80 than in SDS81 at 3 h after salt treatment, whereas higher expression was observed in SDS81 at the remaining time points. Seita.5G412600 displayed a gradual increase in expression throughout the salt treatment, with consistently higher transcript levels in SDS81 compared with SDT80 (**Figure 5A**; **Supplementary Table 6**).

Additionally, Seita.6G011900 and Seita.6G012200, both containing transferase domains, are likely involved in diverse metabolic reactions and substrate conversion processes (**Figure 5B**; **Supplementary Table 6**). Seita.6G011900 showed an expression trend of initial downregulation followed by upregulation, with generally higher expression in SDT80 than in SDS81, whereas Seita.6G012200 exhibited a continuous increase in expression over the course of salt treatment and consistently higher expression levels in SDT80. The gene ID Seita.9G517400, which harbors a CzcO domain associated with metal ion binding and redox homeostasis, was progressively upregulated under salt stress, with overall higher expression levels in SDT80 than in SDS81 (**Figure 5A**; **Supplementary Table 6**).

### 3.6 Comparative analysis of hub gene co-expression networks in Module 5 between two accessions under salt stress

**Figure 6:**
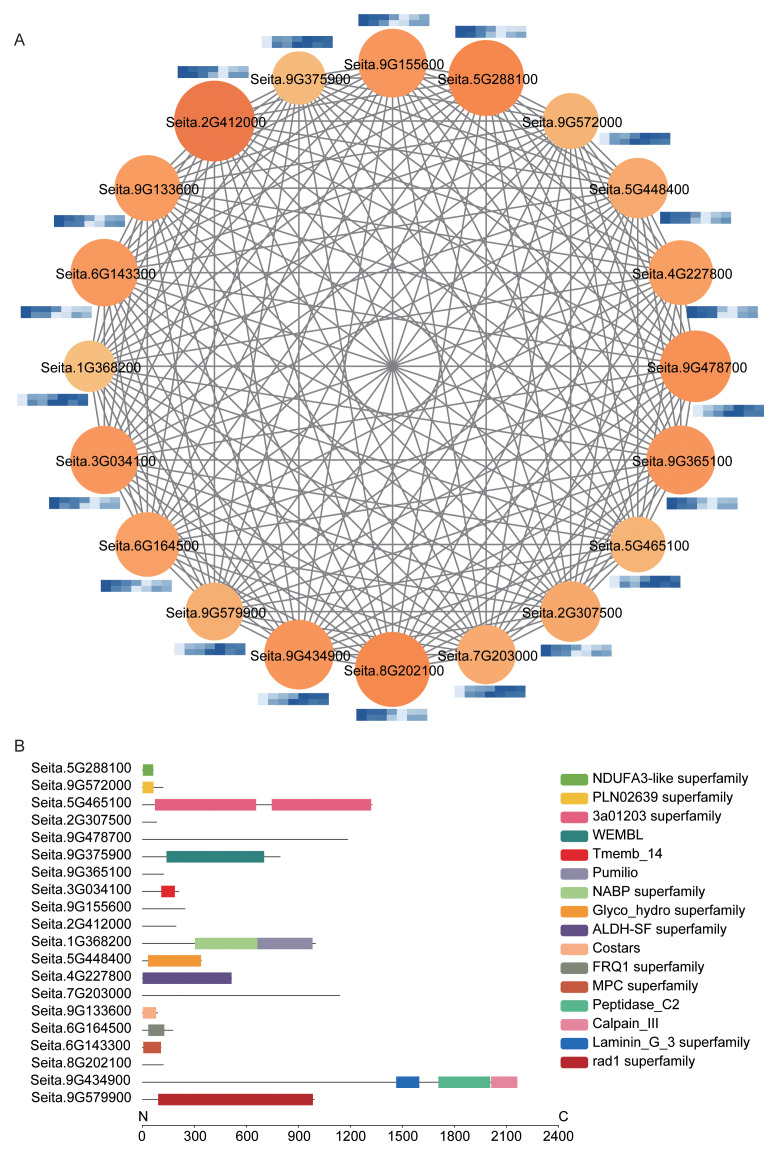
Analysis of hub genes in Module 5. **(A)** Integrated co-expression network and expression heatmap of hub genes. Solid circles represent functional genes, with node size and color reflecting their importance within the network. Heatmaps show min–max normalized and log-transformed expression levels, with the upper and lower rows representing SDT80 and SDS81, respectively, across NaCl treatment at 0 h, 3 h, 6 h, 12 h, 24 h, 48 h, and 72 h, darker blue indicates higher expression. **(B)** Conserved domain analysis of key genes. Lines represent protein sequences, and colored boxes indicate different conserved domains.

Module 5 exhibited highly diverse and complex biological functions, primarily associated with the regulation of protein translation, ROS scavenging, and metabolic reprogramming, with an overall emphasis on metabolic remodeling, detoxification, and energy homeostasis. Domain analysis revealed the presence of the key detoxification-related domain ALDH-SF (Seita.4G227800), encoding an aldehyde dehydrogenase that plays a critical role in eliminating salt stress–induced peroxidation products and in the synthesis of osmoprotective compounds. This gene displayed a similar expression pattern in both accessions, characterized by an initial decrease followed by a subsequent increase during salt treatment (**Figure 6**; **Supplementary Table 7**).

At the post-transcriptional regulatory level, the identification of the Pumilio/NABP domain in Seita.1G368200 suggests that this module may facilitate rapid proteome reorganization by modulating mRNA stability and translational efficiency (**Figure 6B**; **Supplementary Table 7**). Notably, this gene exhibited lower expression in SDT80 than in SDS81 at the early stage of stress, but its expression gradually increased at later stages and exceeded that in SDS81, implying reduced early cellular damage in SDT80 and a delayed activation of protective mechanisms (**Figure 6A**).

In addition, the presence of the calcium signal sensor FRQ1 (Seita.6G164500) and the mitochondrial pyruvate carrier MPC (Seita.6G143300) indicates that Module 5 may contribute to salt stress tolerance by optimizing mitochondrial energy metabolism and activating calcium-mediated stress signaling pathways (**Figure 6B**; **Supplementary Table 7**). Both genes exhibited a trend of initial downregulation followed by a slight recovery under salt stress. Their expression levels were higher in SDT80 than in SDS81 at early time points but became lower at later stages, suggesting a delayed initiation of signal transduction and metabolic adjustment in the salt-sensitive accession (**Figure 6A**).

Furthermore, Seita.5G448400, containing a Glyco_hydro domain associated with cell wall polysaccharide modification, showed a similar expression pattern of initial decline followed by induction (**Figure 6B**; **Supplementary Table 7**). However, its expression level at later stages was lower in SDT80 than in SDS81, indicating that the salt-sensitive accession may require stronger activation of cell wall repair and remodeling processes (**Figure 6A**). Collectively, these results suggest that Module 5 enhances cellular physical and chemical resilience under salt stress through coordinated, multidimensional metabolic reprogramming.

### 3.7 Identification of accession-specific genes through cross-network module comparison

**Figure 7:**
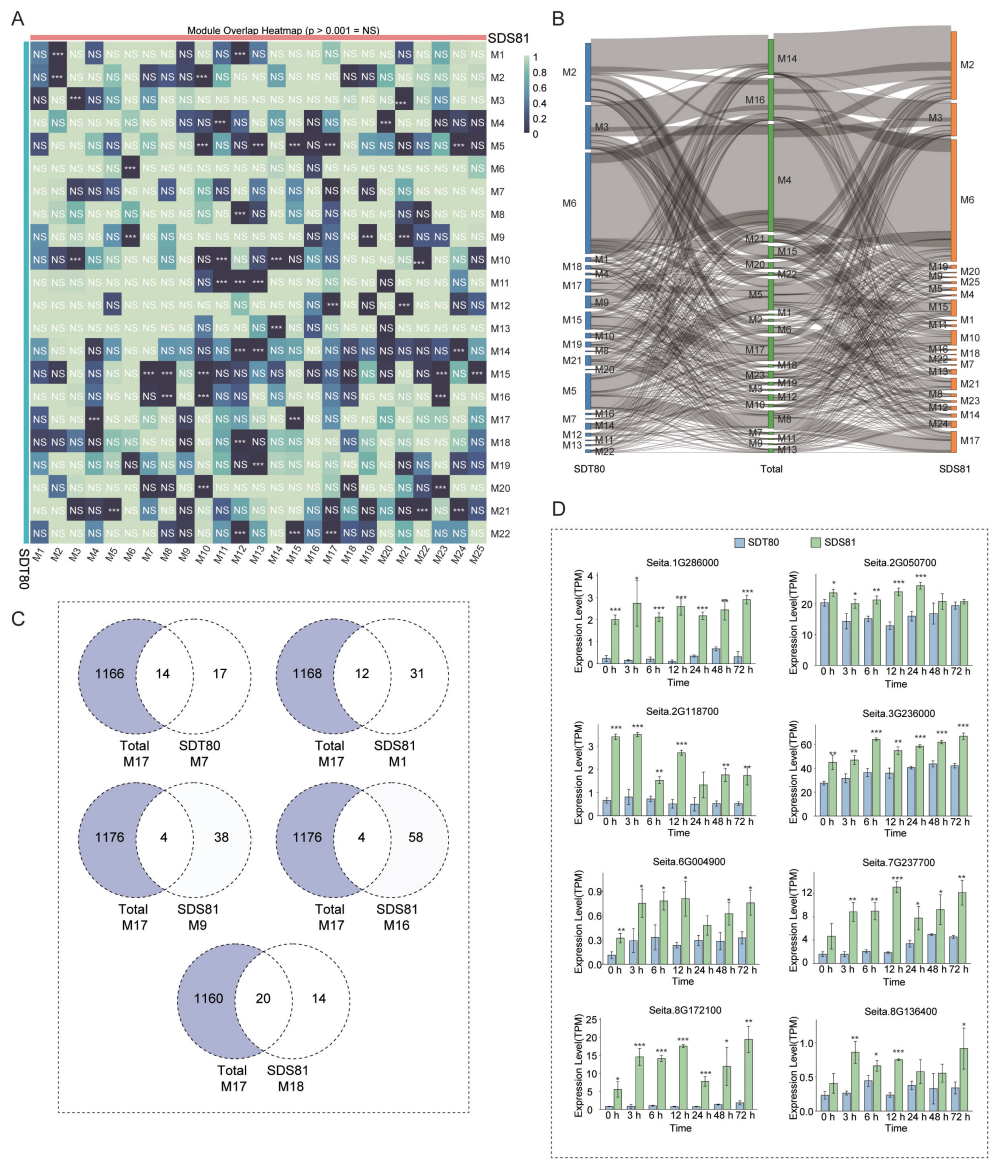
Accession-specific co-expression module analysis. **(A)** Heatmap showing pairwise overlap significance between WGCNA modules derived from SDT80 and SDS81. Color intensity indicates the degree of module overlap, and “NS” denotes non-significant overlap (p > 0.001). **(B)** Sankey diagram illustrating the correspondence of co-expression modules among SDT80, the total dataset, and SDS81. **(C)** Venn diagrams showing the overlap between accession-specific modules and the total dataset module M17. **(D)** Expression profiles of representative candidate genes in SDT80 and SDS81 under salt stress at different time points. Gene expression levels are shown as TPM values (mean ± SD). Asterisks indicate statistically significant differences between accessions (*p < 0.05, **p < 0.01, ***p < 0.001). M, Module.

To validate the reliability of the aforementioned data analyses, WGCNA module analysis was independently performed for the two accessions, SDT80 and SDS81, followed by pairwise comparisons of module overlap (**Figure 7A**). The results showed that both SDT80 and SDS81 possessed accession-specific modules. Specifically, one unique module (M7) was identified in SDT80, whereas four accession-specific modules (M1, M9, M16, and M18) were detected in SDS81. Further examination of the correspondence between each accession-specific network and the total dataset was conducted using Sankey diagrams. These analyses consistently supported the above findings, showing that modules with high overlap exhibited strong connectivity, whereas those with low overlap displayed little to no connectivity (**Figure 7B**).

To identify key genes potentially associated with accession-specific traits, genes from accession-specific modules were intersected with genes from module M17 of the total dataset (**Figure 3C**), which showed the strongest positive correlation with varietal traits, using Venn diagram analysis (**Figure 7C**). Genes shared between each accession-specific module and Total-M17 were defined as accession-specific candidate genes. As a result, 14 shared genes were identified between the SDT80-specific M7 module and total-M17, while 12, 4, 4, and 20 shared genes were detected between the SDS81-specific M1, M9, M16, and M18 modules and total-M17, respectively (**Figure 7C**). These results suggest that these genes may contribute to accession-specific phenotypic variation. Expression analysis of the identified candidate genes revealed that the majority exhibited significantly higher expression levels in SDS81 than in SDT80, a pattern consistent with the results observed for the M17–Variety module shown in **Figure 3C** (**Figure 7D**), further supporting their potential involvement in varietal differentiation.

Based on GO enrichment and functional annotation analyses, seita1G286000, seita2G050700, seita2G118700, and seita3G236000 were found to encode proteins containing valyl-tRNA synthetase, Tesmin/TSO1-like CXC domain, KH domain–containing RNA-binding protein, and scarecrow-like protein 1 domains, respectively. These genes are mainly involved in protein biosynthesis, cell cycle regulation, post-transcriptional regulation, and development-related transcriptional control, playing critical roles in plant growth and tissue differentiation, and may therefore constitute an important genetic basis underlying phenotypic differences among accessions. In contrast, seita6G004900, seita7G237700, and seita8G172100 encode proteins containing phosphorylase kinase–related domains, which may participate in plant stress responses through signal phosphorylation-mediated regulation. Additionally, seita8G136400 encodes a cytochrome P450 protein that is primarily involved in metabolic reaction catalysis and energy metabolism regulation, contributing to the maintenance of metabolic homeostasis. Collectively, these four genes are proposed as key candidate genes underlying the observed differences in stress tolerance among the different accessions.

## 4 Discussion

### 4.1 Ion homeostasis and physiological divergence under salt stress

Salt stress constrains plant growth primarily through ionic toxicity and osmotic imbalance, and the maintenance of ion homeostasis is widely recognized as a central determinant of salt tolerance (Munns and Tester, 2008; Ma et al., 2025). In this study, the salt-tolerant accession SDT80 maintained significantly lower Na^+^ accumulation and a more stable Na^+^/K^+^ ratio than the salt-sensitive accession SDS81 during prolonged salt exposure, indicating more efficient Na^+^ exclusion and/or intracellular sequestration. In contrast, excessive Na^+^ accumulation and disruption of Na^+^/K^+^ balance in SDS81 likely intensified ionic toxicity and compromised cellular functions. Consistently, reduced MDA accumulation in SDT80 suggests alleviated membrane lipid peroxidation and enhanced protection against salt-induced oxidative damage, in agreement with previous observations in salt-tolerant cereal crops (Leng et al., 2025).

### 4.2 Temporal and genotype-specific transcriptomic responses to salt stress

Time-course transcriptome analysis revealed pronounced temporal and genotype-dependent transcriptional reprogramming under salt stress. Both accessions exhibited dynamic DEG patterns, with the largest transcriptional shift occurring at 24 h, suggesting a critical transition from early stress perception to longer-term adaptive or damage-associated responses. Notably, SDT80 showed stronger enrichment of abiotic stress–related functional categories, whereas SDS81 preferentially activated pathways associated with photosynthetic dysfunction, protein degradation, and cell death at later stages. Such contrasting transcriptional strategies suggest that SDT80 rapidly engages adaptive signaling and defense pathways, whereas SDS81 experiences progressive cellular impairment under sustained stress. Similar genotype-dependent temporal differences in salt stress responses have been reported in rice, wheat, and maize (Li et al., 2025; Zhang et al., 2025b).

### 4.3 Modular regulatory architecture revealed by co-expression network analysis

WGCNA further revealed a modular regulatory architecture underlying salt stress responses. Several co-expression modules were strongly associated with accession identity, treatment duration, and physiological traits, highlighting the coordinated regulation of gene networks rather than isolated gene effects. In particular, the Module 14 showed strong positive correlations with treatment duration, Na^+^/K^+^ ratio, and Mg^2+^ content, indicating its involvement in long-term ionic and metabolic adjustment. In contrast, Module 3 was negatively correlated with treatment duration and ion accumulation, suggesting a role in early stress perception and ion homeostasis regulation. The close correspondence between eigengene dynamics and physiological traits supports the biological relevance of these modules and is consistent with previous WGCNA-based studies of abiotic stress adaptation (Wang et al., 2025a).

### 4.4 Functional divergence of key modules underlying salt tolerance

Hub gene analysis revealed clear functional divergence between key regulatory modules. The Module 14 was enriched in transcription factors from the WRKY, MYB, NAC, and tify families, together with calcium- and kinase-mediated signaling components, supporting its role as a central regulatory hub integrating hormonal signaling, stress perception, and metabolic adjustment. These transcription factor families have been widely implicated in coordinating ABA-dependent and ABA-independent stress signaling pathways (Hosseini Pouya et al., 2025). In contrast, Module 5 was dominated by genes involved in detoxification, post-transcriptional regulation, mitochondrial energy metabolism, and cell wall remodeling. The stronger and delayed induction of repair- and protection-related genes in SDS81 likely reflects a compensatory response to more severe cellular damage, whereas SDT80 maintains homeostasis through earlier and more efficient regulatory control. Together, these findings suggest that salt tolerance in foxtail millet is conferred by coordinated, module-level regulation integrating ion homeostasis, stress signaling, and metabolic reprogramming rather than by single-gene effects.

### 4.5 Accession-specific regulatory networks contribute to salt tolerance divergence

By integrating WGCNA based on individual accessions with a combined dataset of both accessions, this study identified distinct co-expression modules and high-confidence candidate genes underlying the divergence in salt tolerance between SDT80 and SDS81. Most candidate genes exhibited higher expression levels in the salt-sensitive accession SDS81, suggesting a compensatory transcriptional response to excessive Na^+^ accumulation, ionic imbalance, and cellular damage, whereas the salt-tolerant SDT80 appears to rely on more efficient physiological regulation and earlier network-level control. Functional annotation indicated that genes involved in protein biosynthesis, cell cycle regulation, post-transcriptional control, and development-related transcriptional regulation may drive accession-specific growth responses under salt stress, while genes encoding phosphorylase kinase–related proteins and cytochrome P450 are likely to participate in phosphorylation-mediated signaling, metabolic regulation, and energy homeostasis. Collectively, these results reinforce the view that salt tolerance in foxtail millet is governed by accession-specific regulatory networks integrating ion homeostasis, stress signaling, and metabolic reprogramming.

## 5 Conclusions

In summary, this study integrates physiological measurements, transcriptome profiling, and co-expression network analysis to elucidate the molecular basis of salt tolerance in foxtail millet. The salt-tolerant accession SDT80 maintains ion homeostasis, limits oxidative damage, and rapidly activates adaptive transcriptional networks under salt stress, whereas the salt-sensitive accession SDS81 exhibits delayed regulation and enhanced stress-induced damage. Co-expression network analysis revealed a modular regulatory architecture, with key gene modules closely associated with physiological traits and stress duration, highlighting the role of coordinated network-level regulation. By integrating accession-specific and combined WGCNA analyses, we identified high-confidence candidate genes involved in growth regulation, stress signaling, and metabolic homeostasis, providing mechanistic insight into the observed phenotypic divergence. Collectively, these findings demonstrate that salt tolerance in foxtail millet is governed by coordinated, accession-specific regulatory networks integrating ion homeostasis, stress signaling, and metabolic reprogramming rather than by single-gene effects, and they offer valuable molecular targets for future functional studies and the genetic improvement of salt tolerance in millet.

## Data Availability

All RNA-seq datasets generated fromNaCl treatments at different time points in this study have been deposited in the CNSA (the CNGB Sequence Archive) project number, CNP0008683.

## Footnotes

1 https://github.com/christophergandrud/networkD3.

2 https://github.com/shenlab-sinai/GeneOverlap.

## Supplementary material

Supplementary Figure 1 GO enrichment analysis of DEGs in SDT80 under salt stress.

Supplementary Figure 2 GO enrichment analysis of DEGs in SDS81 under salt stress.

Supplementary Figure 3 KEGG enrichment analysis of DEGs in SDT80 under salt stress.

Supplementary Figure 4 KEGG enrichment analysis of DEGs in SDS81 under salt stress.

Supplementary Figure 5 Gene expression levels within the co-expression network of Module 3.

Supplementary Figure 6 Gene expression levels within the co-expression network of Module 14.

Supplementary Table 1 Detailed information on RNA sequencing data of foxtail millet samples under salt stress.

Supplementary Table 2 Number of differentially expressed genes (DEGs) identified by pairwise comparisons.

Supplementary Table 3 GO enrichment analysis of differentially expressed genes in key co-expression modules.

Supplementary Table 4 KEGG enrichment analysis of differentially expressed genes in key co-expression modules.

Supplementary Table 5 Protein sequences of key genes within Module 3 co-expression network.

Supplementary Table 6 Protein sequences of key genes within Module 14 co-expression network.

Supplementary Table 7 Protein sequences of key genes within Module 5 co-expression network.
