# Supplementary material for: Differential physiological responses and transcriptome co-expression networks of salt-tolerant and salt-sensitive foxtail millet (Setaria italica (L.)) under salt stress
Source: Front Plant Sci. 2026 Feb 16;17:1772695. doi: 10.3389/fpls.2026.1772695 (PMC12950790; doi:10.3389/fpls.2026.1772695)
Supplement: Supplementary Figure 1 — GO enrichment analysis of DEGs in SDT80 under salt stress. [file Supplementaryfile1.docx]

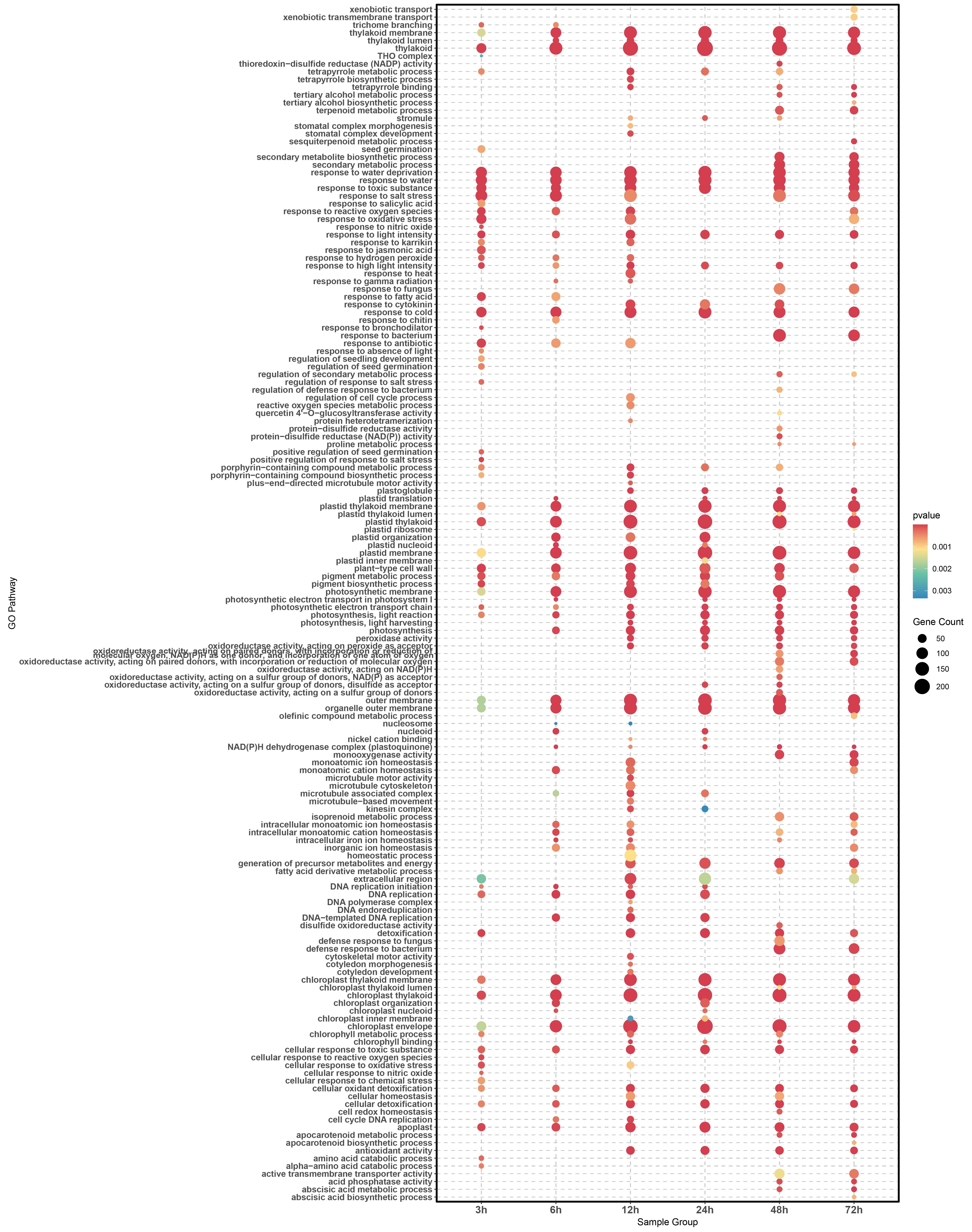


**Supplementary Figure 1 GO enrichment analysis of DEGs in SDT80 under salt stress.**

Dot plot showing the GO enrichment of DEGs in SDT80 at various salt stress time points. Dot size indicates gene count, and color reflects p-value significance, with red representing higher significance.


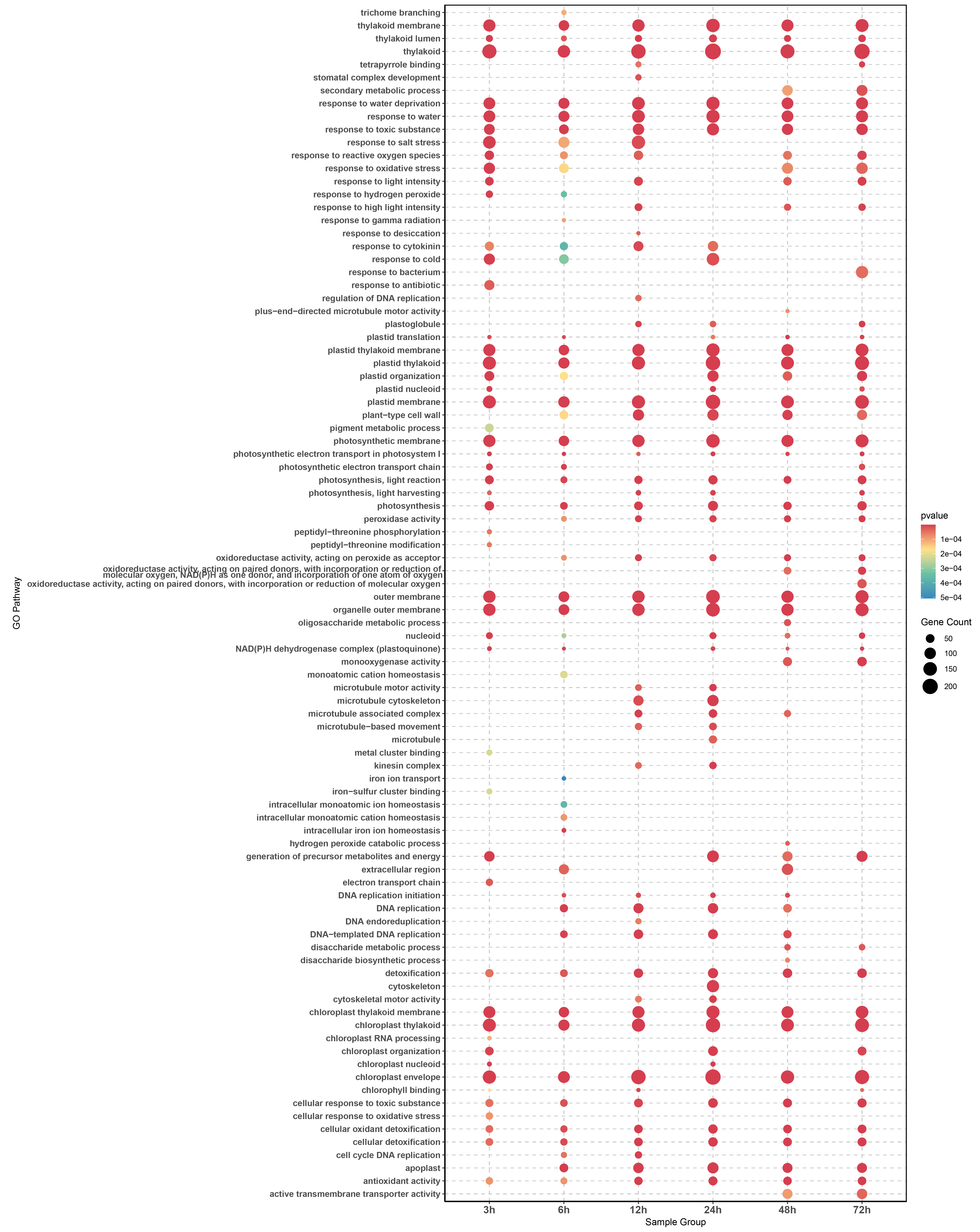


**Supplementary Figure 2 GO enrichment analysis of DEGs in SDS81 under salt stress.**

Dot plot showing the GO enrichment of DEGs in SDS81 at various salt stress time points. Dot size indicates gene count, and color reflects p-value significance, with red representing higher significance.


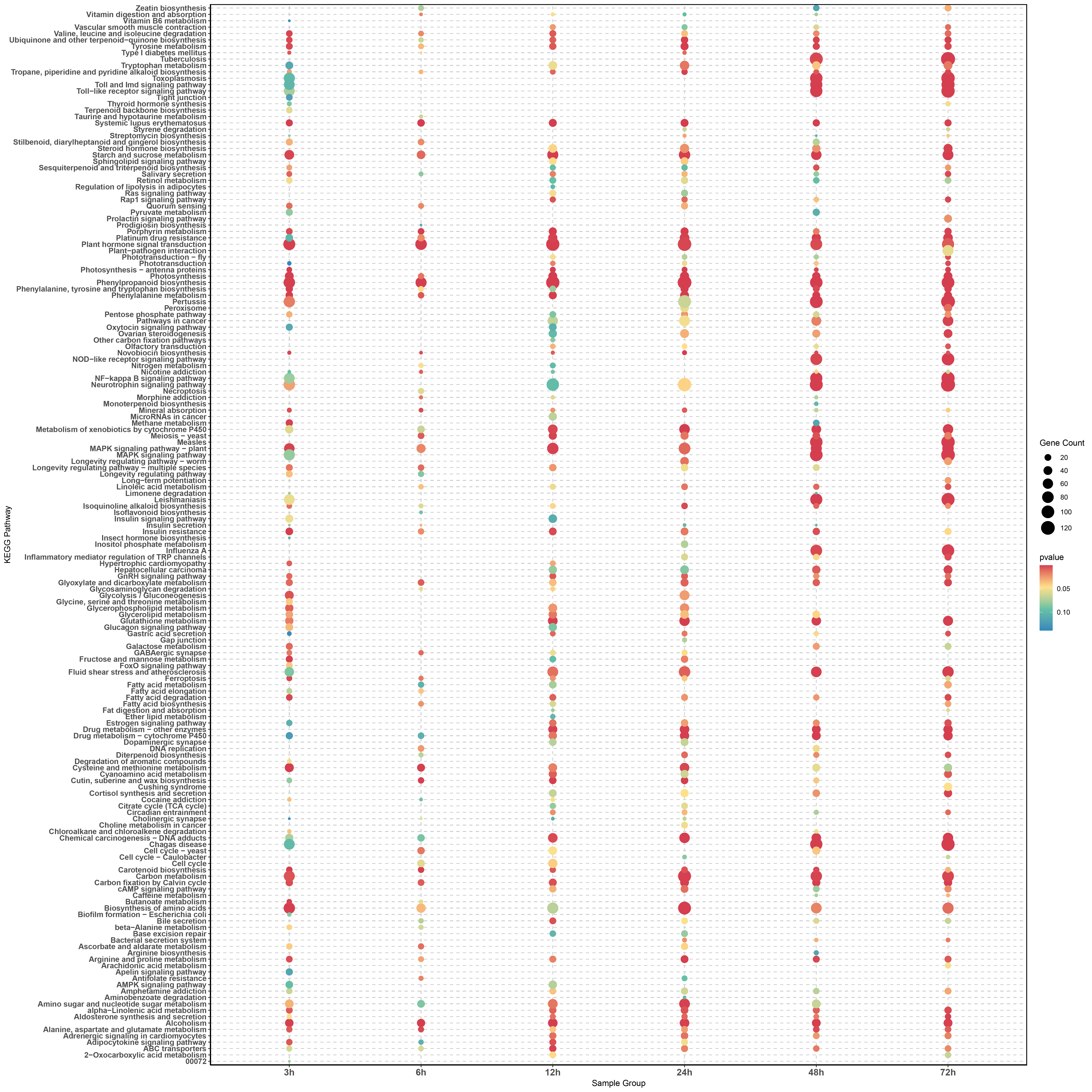


**Supplementary Figure 3 KEGG enrichment analysis of DEGs in SDT80 under salt stress.**

Dot plot showing the KEGG enrichment of DEGs in SDT80 at various salt stress time points. Dot size indicates gene count, and color reflects p-value significance, with red representing higher significance.


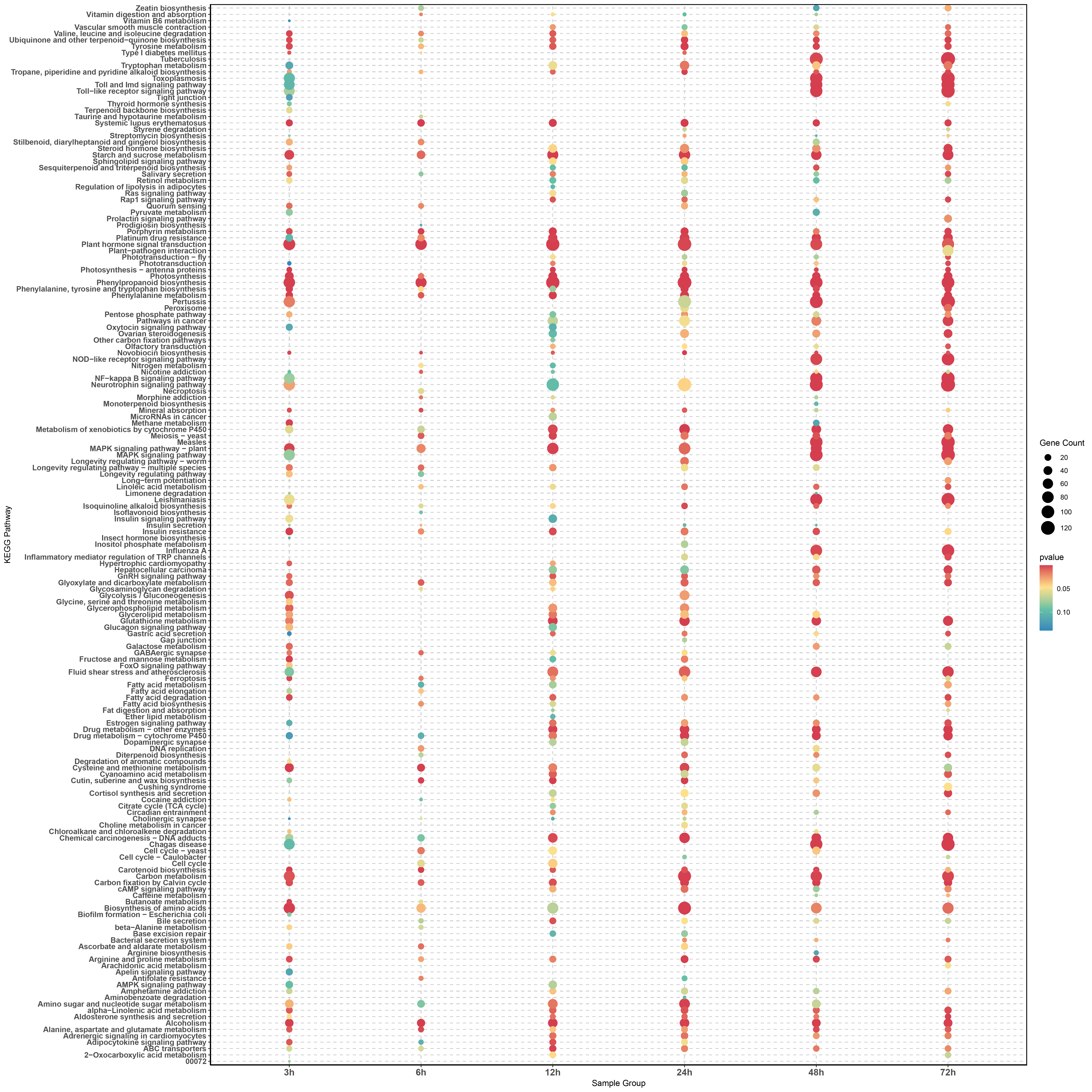


**Supplementary Figure 4 KEGG enrichment analysis of DEGs in SDS81 under salt stress.**

Dot plot showing the KEGG enrichment of DEGs in SDS81 at various salt stress time points. Dot size indicates gene count, and color reflects p-value significance, with red representing higher significance.


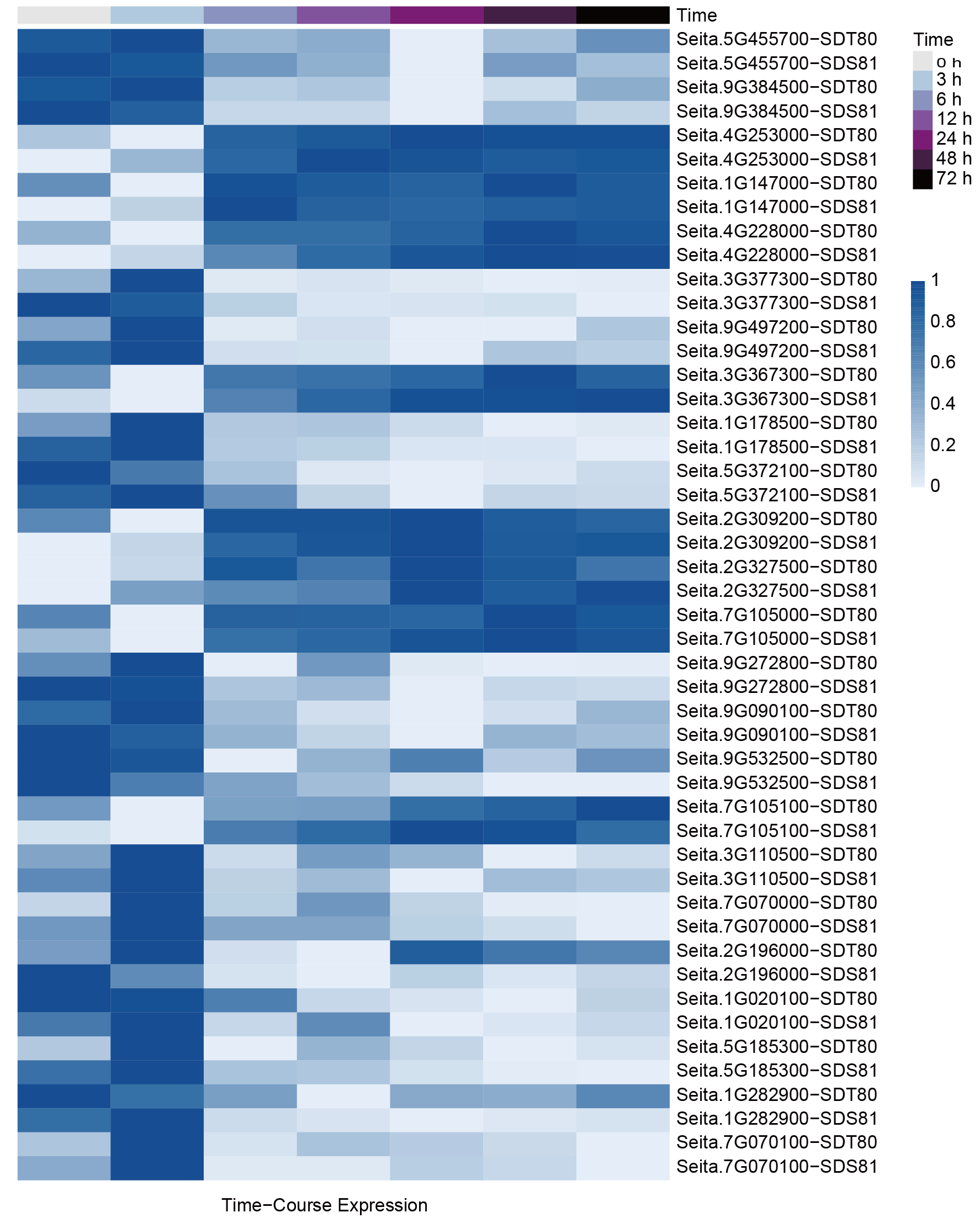


**Supplementary Figure 5 Gene expression levels within the co-expression network of Module 3.** Heatmaps show min–max normalized and log-transformed expression levels, with the upper and lower rows representing SDT80 and SDS81, respectively, across NaCl treatment at 0 h, 3 h, 6 h, 12 h, 24 h, 48 h, and 72 h, darker blue indicates higher expression.


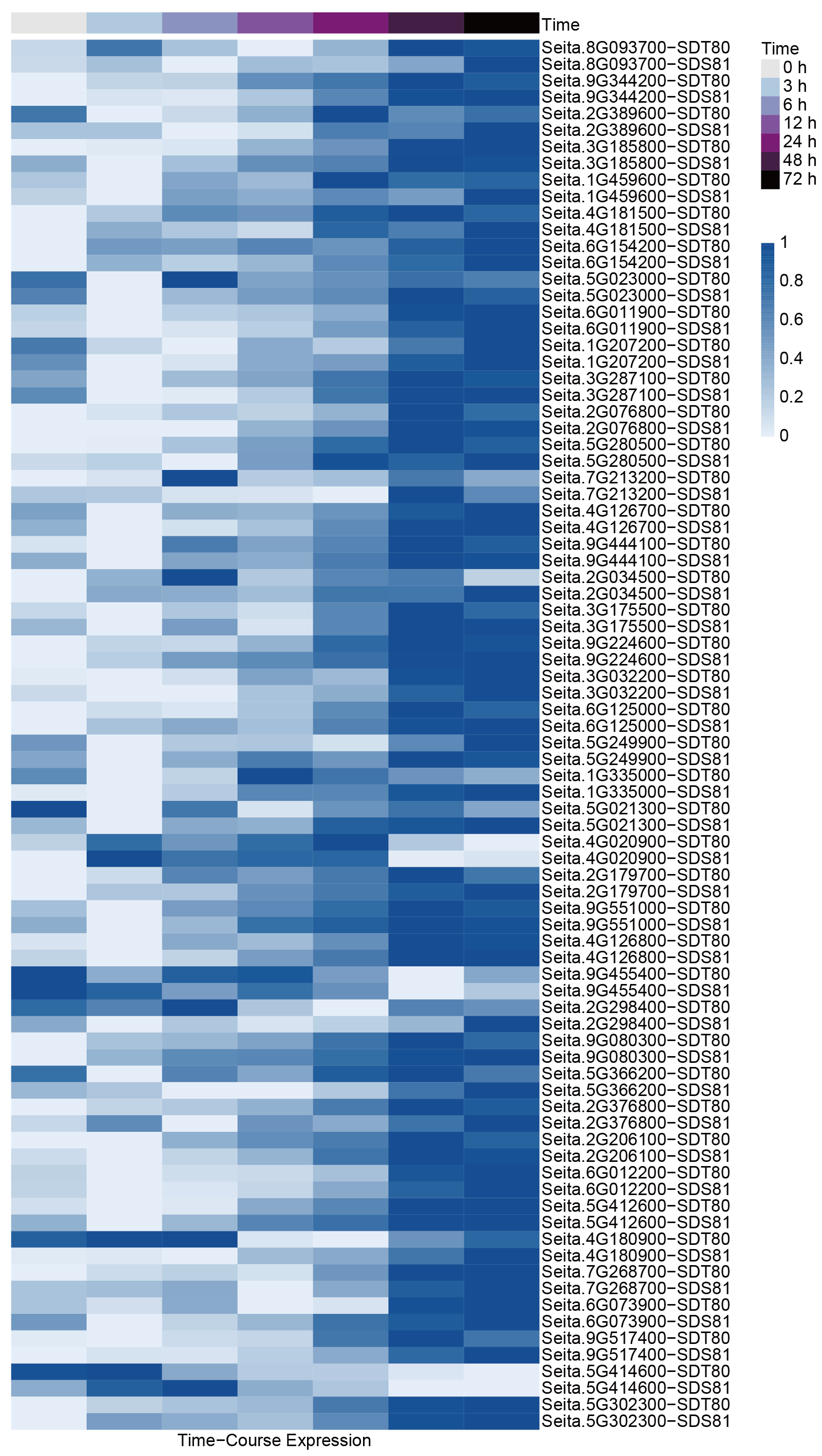


**Supplementary Figure 6 Gene expression levels within the co-expression network of Module 14.** Heatmaps show min–max normalized and log-transformed expression levels, with the upper and lower rows representing SDT80 and SDS81, respectively, across NaCl treatment at 0 h, 3 h, 6 h, 12 h, 24 h, 48 h, and 72 h, darker blue indicates higher expression.
